# Role of biomechanical factors in plaque rupture and erosion: insight from intravascular imaging based computational modeling

**DOI:** 10.1038/s44325-025-00048-8

**Published:** 2025-04-16

**Authors:** Liang Wang, Yanwen Zhu, Chen Zhao, Akiko Maehara, Rui Lv, Xiaoya Guo, Mingming Yang, Gary S. Mintz, Dalin Tang, Haibo Jia, Bo Yu

**Affiliations:** 1 School of Biological Science and Medical Engineering, Southeast University, Nanjing, 210096 China; 2 State Key Laboratory of Frigid Zone Cardiovascular Diseases (SKLFZCD), Key Laboratory of Myocardial Ischemia, Chinese Ministry of Education, Department of Cardiology of the Second Affiliated Hospital, Harbin Medical University, Harbin, 150086 China; 3 Cardiovascular Research Foundation, Columbia University, New York, NY 10022 USA; 4 Department of Cardiovascular Surgery, Shandong Second Provincial General Hospital, Jinan, 250022 China; 5 School of Science, Nanjing University of Posts and Telecommunications, Nanjing, 210023 China; 6 Department of Cardiology, Zhongda Hospital, School of Medicine, Southeast University, Nanjing, 210009 China; 7 Mathematical Sciences Department, Worcester Polytechnic Institute, Worcester, MA 01609 USA

**Keywords:** Acute coronary syndromes, Biophysical methods

## Abstract

Coronary biomechanics including structural wall stress and strain on the vessel wall and flow wall shear stress on the endothelial surface play a vital role in plaque progression, rupture and erosion. This review summarizes recent advances in coronary intravascular imaging, image-based biomechanical modeling, and their applications in investigating possible biomechanical mechanisms of plaque rupture and erosion, and developing interventional therapies targeting unfavorable mechanical conditions for personalized treatment and precision medicine.

## Introduction

Acute coronary syndrome (ACS) is a major cause of morbidity and mortality worldwide, responsible for an estimated 20% of all deaths from cardiovascular diseases (CVD) according to a survey of 122 countries in 2020^1,2^. ACS is characterized by a sudden decrease or block of blood supply to the heart, usually presenting with abrupt onset of a group of critical cardiovascular conditions including non-ST-elevation myocardial infarction (NSTEMI), ST-elevation myocardial infarction (STEMI) and unstable angina^3–5^. The pathophysiological mechanisms of ACS are multifaceted^6^, including non-atherosclerotic related causes^7,8^ and atherosclerotic related causes^9^. Among them, vulnerable plaque rupture and erosion followed by the secondary thrombosis constitute about 55-60% and 30-35% of ACS cases, respectively^10–12^. Understanding these mechanisms to trigger these deleterious plaque behaviors causing ACS is of vital importance in early prevention of ACS, development of novel therapies, and clinical decision-making for interventional cardiologists^13,14^.

**Fig. 1:**
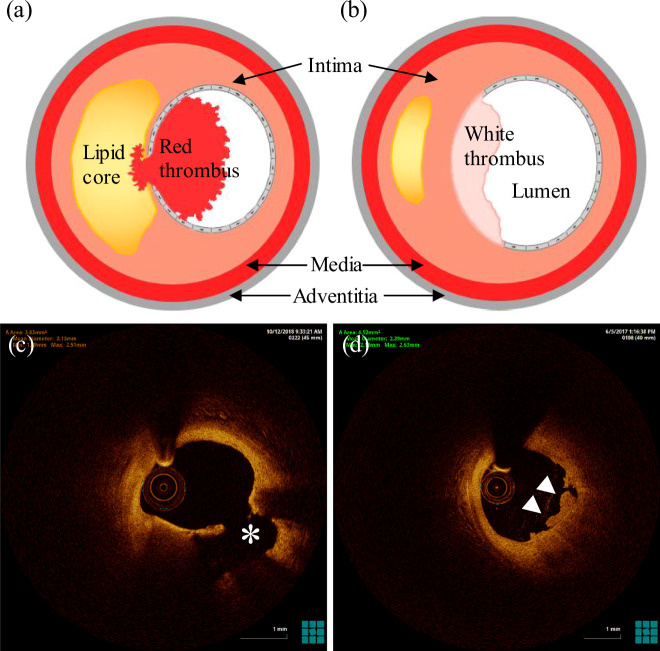
Comparison of morphological characteristics of plaque rupture and erosion to cause ACS. Illustration of cross-sections of ruptured plaque (**a**) and eroded plaque (**b**) with intraluminal thrombosis. In vivo optical coherence tomography images of atherosclerotic plaque rupture with a large emptied cavity (asterisks) communicating with the vessel lumen (**c**), and plaque erosion with a white thrombus rich in platelets (arrowheads) attached at the site of erosion (**d**).

Clinical and autopsy studies have shown that there are dramatic differences in morphological characteristics between ruptured and eroded plaques^15^. Postmortem studies have revealed that plaque rupture is characterized by disrupted thin fibrous cap with luminal thrombus communicating with the underlying necrotic core (Fig. 1(a))^16,17^. The advanced plaque with the morphological characteristics of a large eccentric lipid core and thin fibrous cap infiltrated by macrophages was believed to be the precursor lesion of ruptured plaque, and termed as thin-cap fibroatheromas (TCFA)^18,19^. Compared to plaque rupture, plaque erosion, characterized by loss of the endothelium with overlying platelet-rich thrombus, contains an intact fibrous cap^20,21^. The fibrous cap is a proteoglycans- and hyaluronan-rich matrix with more smooth muscle cells, but less inflammation infiltration (Fig. 1(b))^22,23^. Furthermore, plaque rupture preferentially occurs at the proximal or the most stenotic region of the atherosclerotic lesion in left anterior descending (LAD) artery and right coronary artery, but rarely at the distal region^24–26^. However, most of eroded plaques locate at the distal to the atherosclerotic stenosis and near arterial bifurcation, where disturbed flow prevails^26^. In three major coronary arteries, erosion primarily occurs in the LAD artery, but least likely in left circumflex artery^26–28^. The compositions of the intraluminal thrombus in two cases are also different. In vivo intravascular imaging data indicate that thrombus in erosion tends to be platelet-rich white clot while that in rupture is more fibrin- and erythrocyte-rich^27,29^. These findings suggest that plaque rupture and erosion are two distinct pathophysiological processes with different etiologies^14,22^.

The natural evolution of atherosclerotic plaque including its initiation, progression and eventually rupture and erosion is a complex process, and is greatly influenced by the mechanical forces in the coronary artery^13,16^. Even though progress has been made regarding to morphological and biological characteristics of these plaques, the precise mechanical mechanisms triggering plaque rupture and erosion remain somewhat elusive^30,31^. With considerable advance in the medical imaging technology and computational modeling techniques, it is possible for us to investigate the impacts of mechanical forces on these deleterious plaque behaviors in vivo^32^. In this review, we will summarize the current biomechanical studies on plaque rupture and erosion based on intravascular images, and describe our current understanding of roles of mechanical forces involved in the genesis of these plaque behaviors from computational biomechanical perspectives, to guide the better clinical management of these two mechanisms of ACS for personalized treatment and precision medicine^14,33^.

## Biomechanical forces in coronary artery

**Fig. 2:**
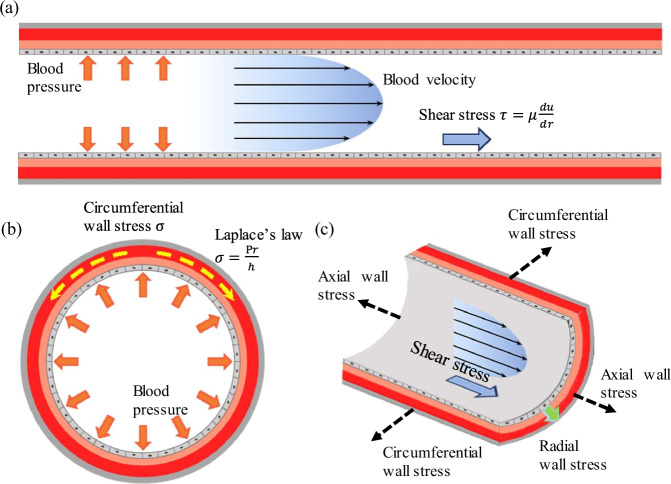
The solid mechanics and hemodynamics exposed to coronary arteries in vivo. **a** Pulsatile blood pressure and flow velocity to induce wall shear stress; **b** Circumferential stress arises from radial expansion and recoil over the cardiac cycle, and Laplace’s law to estimate the wall stress, where r is vessel radius, h is the wall thickness; **c** The wall stress in axial, circumferential and radial directions.

Coronary arteries are constantly subjected to the cyclic biomechanical forces generated by the interactions between pulsatile blood flow and arterial vessel wall motion^13,34^. It is necessary to briefly review the concepts of these biomechanical forces, prior to the understanding of their effects on the coronary plaque. Stress is defined as the force acting on a surface per unit area^35^. There are three main mechanical stresses, that is, the mechanical stresses in circumferential, radial, and axial directions to deform vascular cells, atherosclerotic tissue, and the artery wall (Fig. 2). Circumferential stress is the most commonly investigated stress in the plaque research, mainly arising from the hydrostatic pressure to render the arterial expansion or contraction^36,37^. As a rough estimation, Laplace’s law was used to calculate the circumferential stress under the assumption that coronary artery is a straight thin-wall tube^38^. In normal arteries, circumferential stress is evenly distributed along the radial direction of the vessel wall^39,40^, with average value varying from 100 to 200 kPa^33^, which may increase to around 300 kPa causing plaque rupture in the atherosclerotic artery^36^. Similar to stress, strain is defined as the amount of deformation by a structure as a consequence of an external applied force^38^. In the one-dimensional situation, strain reflects changes in the length of the material over the initial length. In atherosclerotic arteries, the plaque exhibits higher strain distribution, which is the consistent with the site of plaque rupture^38^.

Wall shear stress (WSS) is a measure of tangential frictional force per unit area acting on the endothelial surface by blood flow^41^. In a simple case of laminar flow in a rigid cylinder tube, the magnitude of WSS is directly proportional to mean flow velocity and plasma viscosity, but inversely proportional to vessel diameter according to Poiseuille flow^42^. In the realistic situation, the magnitude of WSS relies on the arterial wall motion, blood flow velocity, and plasma viscosity^34^, and is very sensitive to the local coronary artery geometry^41^. Average WSS value varies from 1 to 7 Pa (corresponding to 10 to 70 dynes/cm^2^) in the arterial vascular system under the physiological conditions^43^. However, the hemodynamic conditions were disturbed at some anatomical sites, such as outer wall of bifurcations and inner curved wall of arterial segments^44^, inducing low WSS. Early atherosclerotic plaques preferentially form in these regions as a result of flow-induced endothelial injury and arterial inflammation^43^. Other hemodynamic factors, such as time-averaged wall shear stress, and oscillatory shear index among others, are also investigated to influence the plaque initiation and development, but they are less explored in the plaque rupture and erosion studies. Comprehensive reviews of these hemodynamic factors could be found in previous excellent reviews^41,45^.

Decades of investigations have demonstrated the critical role of biomechanical forces during the arterial physiology or atherosclerotic pathology^13^. Unfortunately, these biomechanical stresses have been difficult to measure experimentally under in vivo state, putting a barrier to thoroughly understand the function of the wall stress and shear stress^46,47^. With the rapid advances in intravascular imaging, image-based computational models of the coronary arteries could incorporate all the relevant information to recapitulate these mechanical forces in a patient-specific setting, and examine their association with the plaque behaviors, particularly plaque rupture and erosion^30,38^.

## Intravascular imaging

Historically, the morphological descriptions of atherosclerotic lesions underlying acute coronary syndromes were originally provided by autopsy studies^16,48^. They provided detailed morphological information to distinguish different types of plaque substrates related to ACS including plaque rupture^49^, plaque erosion^26^, and calcified nodule^50^, which have established the foundation for the in vivo diagnosis of the plaques in clinical setting^51^. For imaging plaque morphology in vivo, intravascular ultrasound (IVUS) and optical coherence tomography (OCT) exhibit superior performances over other noninvasive imaging technologies in identifying these plaque types, as they could reliably detect the vessel boundaries and various plaque compositions^52,53^. IVUS can visualize deep vessel wall structure with a penetration depth of 4-8 mm, whereas OCT can only provide superficial structures because the penetration depth of light into the vessel wall is about 1-2 mm^54^. However, with its 100–200 μm resolution, IVUS cannot reliably detect vulnerable plaques with thin cap thickness (threshold value 65 μm). OCT, given its superior imaging resolution (10-20 μm), possesses higher ability in visualizing the luminal surface of the vessel and the microstructure of atherosclerotic plaque such as fibrous cap, thrombus, and calcifications^55–57^. These advantages make it the most suitable imaging modality to assess plaque erosion and fibrous cap disruption in plaque rupture^58,59^. In a retrospective study based on 112 patients with STEMI who had undergone both OCT and IVUS, IVUS could only detect 17 plaque rupture sites among the 72 OCT-detected plaque rupture^60^.

Recently, a commonly accepted classification scheme has been proposed by Jia et al.^51^ to classify three pathological substrates causing ACS based on in vivo OCT images. In this scheme, plaque rupture was defined as a discontinuity in the fibrous cap with a communication between lumen and a plaque cavity formed due to the loss of lipid-rich necrotic core (Fig. 1(c))^61^. Plaque erosion could be determined if a visualized plaque with intact fibrous cap underlying a luminal thrombus is present (Fig. 1(d))^62^. A plaque with irregular luminal surface without the presence of any thrombus, or attenuation of underlying plaque by thrombus and no superficial lipid or calcification immediately adjacent to the thrombus site were also defined as probable erosion^51^. This could serve as a basis to reconstruct image-based computational models to simulate the coronary biomechanics in vivo to investigate their impact on plaque behaviors^11,63,64^.

## Image-based biomechanical modeling

The theoretical basis of the computational simulation is that coronary biomechanical stresses from the solid and fluid motion could be accurately described by the mathematical equations, and solved by finite element approach^33,34^. In vivo medical image-based computational models have been employed to simulate the biomechanical stresses in both coronary plaques-wall and blood flow, to investigate the mechanical mechanisms of coronary atherosclerosis^46,47^. The computational models could be categorized into three types with their advantages and limitations provided in the previous references^46,54^: 1) Computational fluid dynamics (CFD) model only considers the motion of the blood flow, and uses Navier-Stokes equations as the governing equations to simulate wall shear stress, fluid velocity, pressure, and other hemodynamic factors^41^. 2) Computational solid mechanics model only considers the coronary plaque wall in the model. The governing equation is much more complicated, including the motion equation, displacement-strain relationship, and constitutive equation of the coronary wall tissues, which can be solved to obtain the stress and strain conditions in the artery wall^65^. As part of the governing equations, the mechanical properties of coronary wall and plaque compositions described by constitutive equations have a profound effect on fully recapitulating the biomechanical conditions within the blood vessel^34,66^, which in turn play a crucial role in shaping plaque behaviors, like rupture and erosion^67,68^. 3) Lastly, fluid-structure interaction (FSI) model combines both fluid and solid models, and allows the simultaneous analysis of solid and fluid domains to derive the comprehensive biomechanical conditions in the coronary artery wall and blood flow^69^. Currently, most computational models of coronary plaques, especially solid and FSI models are created based on IVUS and OCT images as they can accurately reconstruct the vessel wall geometry with multiple plaque components^70,71^.

For computational models, the following essential elements need to be considered to accurately calculate biomechanical forces in the coronary plaque: a) coronary geometry reconstruction from patient-specific images, ; b) vessel and plaque component material properties; c) complete set of structure and/or flow governing equations (equation of motion, equation of continuity, structural stress/strain relationship, strain-displacement relationship); d) specified boundary conditions; e) initial conditions and residual stress/strain of the blood vessel^69^. Patient-specific coronary geometry was reconstructed by integrating IVUS or OCT images with angiography image to recover the vessel shape in three-dimensional space. However, this coronary geometry represents its in vivo state when the vessel is physiologically pressurized and longitudinally stretched^69^. Typically, an inverse process was conducted to find the zero-pressure geometry as the initial geometry for numerical simulation^69,72^. Under given boundary conditions, the blood would flow, and the vessel wall would start deforming in response to the external forces prescribed. Some computational studies even consider the coronary movement caused by cardiac contraction to accurately simulate the biomechanical conditions^73^. More details on the model construction process could be found in the previous comprehensive references^46,54,69^.

## Role of biomechanical stresses in vulnerable plaque research

### Mechanical factors in vulnerable plaque development and progression

Mechanical forces influence multiple aspects of vascular physiology and function, and are crucial in the pathological development of coronary atherosclerosis^13,16^. There are various types of atherosclerotic plaques, ranging from early lesions of atherosclerosis consisting of the adaptive lesion with fatty streak and fibrous tissue, to advanced complicated lesions^16,19^. Before the occurrence of critical behaviors like rupture and erosion, the plaques undergo a destabilization process, transforming into more advanced plaques with vulnerable features^16^. Computational studies have performed to uncover the complex relationship between the biomechanical factors and plaque progression.

In vitro experimental studies and clinical observations both reported that the plaque initiates at the location where low and oscillating shear stress usually occurs^74,75^. Many clinical studies have showed that the conclusion is also true for advanced plaque progression^76^. In the PREDICTION study with a large patient pool (*n* = 506), Stone et al. constructed CFD models based on IVUS image to demonstrate that low WSS (< 1.26 Pa) at baseline could predict plaque burden increase during 1-year follow-up period^77^. In another CFD study involving 20 patients with IVUS follow-up data, Samady et al. found that vessel cross-sections with baseline WSS below 1.0 Pa showed increased plaque area at follow-up (*p* = 0.027)^78^. Bourantas et al. further reported that low WSS and plaque burden were significant predictors of disease progression, defined as simultaneous lumen reduction and plaque burden increase^79^. They noted that combining WSS with plaque characteristics could achieve a prediction accuracy of around 85%, surpassing the use of plaque characteristics alone. Additionally, Liu et al. and other researchers have reached similar conclusions, showing that lesion sites with low WSS are linked to plaque growth^80,81^.

Besides shear stress, wall stress was also considered to aid in predicting plaque progression behaviors. By performing computational solid mechanics for 8182 virtual histology-IVUS frames from 101 plaque lesions, Costopoulos et al. found that areas with high wall stress was associated with larger plaque burden decrease at follow-up, compared to areas with low wall stress^82^. Another study by Wang et al, have demonstrated that integrating all morphological and biomechanical factors provided better results in predicting plaque progression^83^. Even though more prediction studies have echoed this finding, some researchers claimed that incorporating biomechanical factors into predictive models only yields marginal improvements in prediction accuracy (~ 5–10%)^79,84,85^. Furthermore, these prediction studies did not clearly show quantitative relationship between wall stress and plaque progression. Further mechanistic researches are warranted to validate these clinical observations via finding a proper animal model or other in vitro models for plaque rupture^86,87^.

### Mechanical factors in plaque rupture

#### TCFA and plaque rupture

Pathological studies claimed that TCFAs were the precursor lesions prone to rupture^18^. However, large clinical studies, such as the PROSPECT and VIVA studies observed that only 5% of the identified TCFAs would undergo plaque rupture during a 3-year follow-up period, suggesting that plaque morphology alone cannot accurately predict future plaque rupture^88,89^. Biomechanical factors are considered as important cues triggering plaque rupture, so they might aid in identifying vulnerable plaques to rupture^13^.

#### Wall stress in plaque rupture

Mechanically specking, the determinants of plaque rupture lie in two aspects: intrinsic plaque vulnerability predisposes it to rupture, and extrinsic forces, such as biomechanical and hemodynamic stresses precipitate rupture^17^. When local wall stress within an atherosclerotic lesion exceeds its material strength, the plaque would disrupt^34^. These plaque disruptions are typically located in the fibrous cap or the shoulder region^16^, often co-localizing with the sites of maximum wall stress^36^. The material strength of the fibrous cap or shoulder region was determined by the integrated material strength and interactions of plaque compositions, such as collagen fiber, macrophage, and microcalcification in the plaque site^67^. Animal experimental studies have shown that excessive collagen breakdown combined with inadequate synthesis weakens plaques, thereby rendering these sites prone to rupture^90,91^. Computational studies also have provided extensive evidence to confirm the conclusion. The first finite element analysis of the plaque rupture was performed by Peterson et al^92^. based on histological images. The simulation results showed that high wall stress was found on the fibrous cap area, especially near the edge of the plaque cap, correlating well with the site of intimal tear at necropsy. This seminal study inspired the finite element models in studying the biomechanical mechanisms of plaque progression and rupture^13^. More quantitative analysis showed that the local maxima of wall stress normally exceeds 2250 mmHg ( ~ 300 kPa) for the ruptured plaques, a well-accepted threshold value for assessing plaque rupture risk from the biomechanical perspective^36^. It is worth noting in these studies using ex vivo histological images, that the post-rupture state of the plaque geometry from the histological section was repaired to reconstruct the pre-rupture geometry to investigate the biomechanical stress driving the rupture behavior^36,92^.

Computational studies based on in vivo images from patient-specific data also support that rupture risk was positively associated with the wall stress value^93,94^. In a comparison study with OCT images from 10 ACS patients and 10 stable patients, the wall stress values from the ruptured plaques were significantly higher than those from stable plaques^93^. The observation was also found in a similar study by performing 3D FSI models^94^. In vivo image based finite element analysis also established the impacts of multiple key morphological features on the stress calculation. As the fibrous cap thinner, the mechanical stress conditions would be higher, and the lipid component had a significant positive correlation with stress values^95^. All these analyses consistently conclude that higher wall stress is an important factor in triggering the rupture behavior of coronary plaques. Thus, biomechanical stress could therefore potentially act as a useful indicator for rupture risk assessment^96^. Besides, the mechanical stress would also influence the fibrous cap strength via mechanotransduction pathways^13^. The plaque areas experiencing high mechanical stress are often infiltrated by monocytes and macrophages to overexpress matrix metalloproteinases (MMPs)^97^. These MMPs are crucial in destabilizing the lesion though degradation and weakening of the collagenous extracellular matrix, posing a higher risk for plaque rupture^98^.

#### Wall shear stress in plaque rupture

Although the impact of wall stress on plaque rupture is rather established, the association between wall shear stress and rupture has not been studied extensively^42^. Many evidences have shown that the rupture occurs at the locations with elevated WSS^99^. In patients with ACS, it is observed that high shear stress is normally at the upstream plaque shoulder where rupture often occurs. Therefore, it has been postulated hat high shear stress might be the primary contributing factor^100^. In vivo evidence from a CFD analysis based on IVUS image from 20 patients with ruptured plaques, showed that local elevation of blood pressure and shear stress could be observed at the rupture sites on each plaque surface^101^. A possible mechanobiological explanation is that a observational studies reported high shear stress to activate MMPs and promote the fibrous cap thinning^102^ and eccentric remodeling^103^ in an arteriovenous fistula model. If a similar process occurs in advanced atherosclerotic lesions, it could explain the presence of a thin fibrous cap prone to rupture in high shear regions. Additionally, reduction in smooth muscle cell proliferation and increase in cell death were also reported at the local site with elevated WSS^104^. This would result in reduced extracellular matrix synthesis, responsible for potential plaque rupture.

**Fig. 3:**
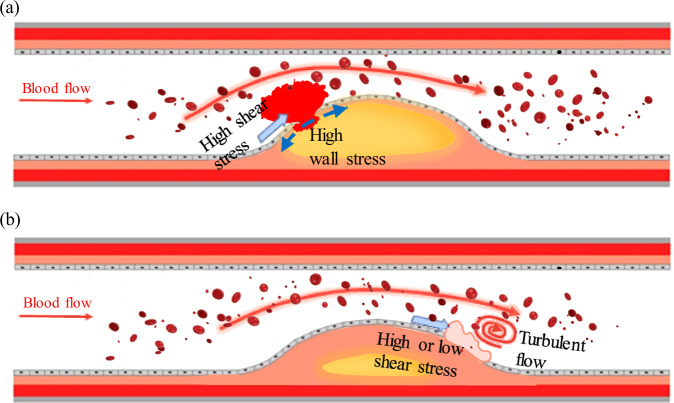
Schematic representation of the differences in morphological characteristics and biomechanical factors in plaque rupture (**a**) and erosion (**b**).

Given both solid mechanics and hemodynamics greatly influenced the rupture of atherosclerotic plaques, a unifying theory was proposed by Pedrigi et al. that co-localization of high wall stress and shear stress-induced plaque weakening will finally lead to plaque rupture (Fig. 3(a))^32^. This hypothesized theory may explain the observation that only the minority of TCFAs lead to ACS. Still, further experimental and clinical imaging studies are needed to investigate the co-localization of these biomechanical parameters in identifying rupture-prone TCFAs.

### Mechanical factors in plaque erosion

Compared to plaque rupture, plaque erosion is a recent emerging mechanism causing ACS. Therefore, fewer studies were performed on the mechanical factor inducing plaque erosion^105^. Wall shear stress, as the most studied mechanical factor, has been proposed as a driver of plaque erosion by promoting loss of endothelium. Early animal studies have yielded contradictory findings regarding the relationship between shear stress and the reduction of endothelial cells. Tricot et al. collected human carotid atherosclerotic plaques that exhibited endothelial apoptosis, as evidenced by immunostaining^106^. Their quantitative analysis showed a consistent preference for endothelial apoptosis in the downstream of the plaques, where low shear stress was supposed to be prevalent. Further animal studies have confirmed that the endothelial apoptosis would lead to the endothelial erosion and vessel thrombosis^107^. Rather quantifying the WSS magnitude, Sumi et al. qualitatively described that disturbed blood flow and oscillatory shear stress occurring after stenosis resulted in erosive damage and thrombus formation on the smooth muscle cell-rich neointima^108^. While these studies suggest a correlation between low WSS and endothelial cell apoptosis, a study using a rabbit model reported that increased WSS could lead to endothelial cell detachment and thrombus formation in areas rich in smooth muscle cells within atherosclerotic plaques^109^.

The inconsistent conclusion also persists in clinical studies in vivo by performing CFD analysis based on medical images (Fig. 3(b)). Table 1 lists the computational studies to investigate the mechanical driver of plaque erosion. Unfortunately, these studies also do not draw a firm conclusion^105^. In a case report by computing WSS from OCT based CFD analysis, Giannopoulos et al. demonstrated the co-localization of plaque erosion and low WSS in one patient involving ACS^110^. In contrast, Vergallo et al. reported a case where high WSS occurred at a location with future plaque erosion and luminal narrowing confirmed by OCT scan at 4-year follow-up^111^. Based on Table 1, the majority of CFD analysis reported that high WSS and WSS gradient values might the critical triggers for the onset of plaque erosion. Hakim et al. demonstrated that eroded plaques had significantly higher WSS and WSS gradient than stable ones^112^. Yamamoto et al. and M McElroy et al. also found that WSS and WSS gradient were higher at the plaque erosion sites compared to non-erosion sites for most of the patients examined^26,113^. Continued analysis showed that elevated WSS and WSS gradient at the erosion site persisted up to 12 months if no coronary stent was performed^105^. Except for the low and high WSS theories, one exceptional study by Campbell et al. indicated no association between high or low WSS values at plaque erosion sites^114^. The inconclusive of this study might be partially due to the small patient size (n = 3). Another reason may be that their coronary geometry was reconstructed from angiography by assuming an elliptical cross-section of vessel lumen. This assumption ignored some local coronary geometry variation, which great influenced local WSS values^41^. Even if most studies favor the high or low WSS theory, there are no large-scale studies with enough patient number to come to a definite conclusion with strong statistical power.

**Table 1:** Image based CFD studies of plaque erosion

Ref.	Imaging Modality	Sample Size	Main findings/Conclusion (Key Risk Factors, Prediction Accuracy)
Campbell et al. 2013^114^	Angiogram	3 PE	Neither high nor low magnitudes of mean WSS were associated with sites of plaque erosion. Oscillatory shear index (OSI) and local curvature were also not associated with erosion.
Giannopoulos et al. 2016^110^	OCT	1 PE	Pre-erosion flow simulation revealed an area of low WSS and blood velocity at the downstream shoulder, co-localizing with the site of erosion and thrombus.
Vergallo et al. 2019^111^	OCT	1 PE	Plaque erosion co-localized with the area exposed to high WSS after percutaneous coronary intervention.
Yamamoto et al. 2019^26^	OCT	18 PE	Plaque erosion and thrombus occurred at the area of peak WSS and WSS gradient in 17 of 18 lesions.
McElroy et al. 2021^113^	OCT	17 PE	The sites of adherent thrombi (assumed to be synonymous of endothelial erosion) had significantly increased time averaged WSS, maximum WSS, time averaged WSS gradient with a reduction in relative residence time, compared to a non‑diseased reference segment.
Thondapu et al. 2021^63^	OCT	19 PR 18 PE	High WSS gradient is independently associated with plaque rupture while high WSS gradient, WSS, and oscillatory shear index associate with plaque erosion.
Kim et al. 2022^105^	Follow-up OCT	23 PE at baseline	WSS and WSS gradient values were higher at the plaque erosion sites compared to non-erosion sites. Elevated ESS and ESSG at the erosion site persisted up to 12 months.
Russo et al. 2023^64^	OCT	11 PE, 14 PR, 24 stable plaques	Plaque rupture exhibited a significantly higher WSS, with plaque erosion having an intermediate WSS value between stable and rupture plaques.
Hakim et al. 2023^112^	OCT	24 PE; 22 stable plaques;	Plaque erosion was strongly associated with higher WSS, WSS gradients, and plaque slope as compared with stable plaques.

*PR* plaque rupture, *PE* plaque erosion

#### Wall stress and strain in plaque erosion

Due to the scarce of solid mechanical analysis, the relationship between plaque erosion and wall stress/strain has been rarely explored. To date, the sole study specifically addressing mechanical stress and strain for the onset of plaque erosion was conducted by Campbell and colleagues^65^. Their findings indicated no association between mechanical strain and plaque erosion, suggesting solid mechanics may not play a significant role in driving late-stage erosion-related complications.

## Clinical perspectives

### Integrative Perspective of Atherosclerotic Plaque Researches

Clinical diagnosis and management of coronary atherosclerotic diseases mainly relies on the plaque morphological characteristics. However, these morphological-information alone are not enough to provide accurate identification of culprit lesion for adverse coronary events^13,89^. For example, only 5% of the identified TCFAs are associated with ACS events caused by plaque rupture, implying that plaque morphology is not sufficient to predict plaque rupture^13,88,89^. As biomechanical factors are involved in plaque development, they might help to identify vulnerable plaques predispose to future coronary events. Therefore, it is necessary to integrating morphological and biomechanical factors for better understanding the plaque behaviors, including plaque rupture and erosion^54,83^.

This integrative approach was adopted in prior studies to reveal possible mechanical relationship with plaque progression, and destabilization process^77,78^, which would shed light on guiding the plaque risk assessment using these biomechanical stresses. The PREDICTION study found that low shear stress independently predicted luminal obstruction in patients with ACS^77^. Although clinical event rates were too low to sufficiently evaluate the effects of shear stress on clinical outcome^77^, the study suggests that biomechanical factors could be relevant in assessing progression-prone areas. In solid mechanics, a MRI-based study has proposed a scheme on carotid plaque biomechanical stress to classify the plaque vulnerability to rupture^96^. As data accumulated enough, the biomechanical wall stress hold the potential to rate the rupture risk of atherosclerotic plaques. Currently some threshold values of biomechanical stress have been proposed, however, detailed schemes on grading plaque vulnerability using these biomechanical factors have not been proposed yet based on in vivo image data^36,96^. This is also the case for plaque erosion. Given their close association, WSS may hold the potential for the risk stratification of future plaque erosion. And it can be imagined that the biomechanical factors would also integrate with morphological features for predicting the onset of the plaque erosion.

Machine learning or other artificial intelligence methods could also be adopted in this integrative approach to include more risk factors, not just morphological and biomechanical factors to assess future plaque behaviors, particularly when a large volume of data is being analyzed^115–117^. Based on 19826 patients with ACS, PRAISE study group have demonstrated the feasibility and effectiveness of machine learning-based approaches to predicting all-cause death, recurrent acute myocardial infarction, and major bleeding after acute coronary syndrome^115^. Koloi et al. also shown that machine learning methods can accurately predict early-stage coronary artery disease using a set of clinical characteristics and routine laboratory markers^116^. These reports demonstrate the strong capabilities of machine learning and artificial intelligence in predicting the behavior of atherosclerotic plaques, and applications of these methods should be recommended in future research.

### Diagnostic and prognostic implications of image-based biomechanical models

Biomechanical modeling has been increasingly employed in the diagnosis, clinical decision-making, and treatment for patients with coronary artery disease^115,118^. A notable example is fractional flow reserve (FFR) in coronary atherosclerotic lesions, which reflects the pressure drop from the proximal aorta to the coronary segment distal to the lesion during maximal vasodilation^118,119^. According to the latest ESC guidelines, percutaneous coronary intervention (PCI) is recommended when FFR is ≤0.8^120^. Large clinical trials have shown that FFR-guided PCI achieves superior follow-up outcomes in coronary revascularization compared to angiogram-guided PCI^121^. CFD techniques based on coronary angiography have been demonstrated to be able to accurately calculate FFR in patients with stable angina, offering a less non-invasive alternative to catheter-based measurements for guiding PCI^118^. Another study also shown that CFD models can be used to simulate various potential surgical plans on treatment of intracranial aneurysms to determine the optimal one for a given patient^122^.

An important potential clinical application of solid mechanics would be identifying sites exposed to excessive forces associated with high plaque rupture risk^34,36,96^. Based on computational modeling, these studies have revealed a positive relationship between wall stress and plaque rupture risk^36,95^. Still, a diagnostic criterion based on wall stress to stratify the rupture risk should be established, and further should be extensively validated through large-scale clinical trials. With firm conclusion can be draw on the relationship between the plaque behaviors and these biomechanical stresses, the biomechanical indicators could revolutionize the triage and management of patients presenting with ACS^13^. Such an advancement would represent a step towards a more personalized approach, and illustrate the value of translation of basic science insights to clinical practice.

### Biomechanical modulation in plaque rupture and erosion treatment

The biomechanics of plaque are not only relevant to the development of plaque rupture and erosion, but also should be considered when explaining the mechanistic role in their treatment. Effective clinical treatment option could be specifically explained by modulating the biomechanical conditions in the coronary plaques. Here are two simple examples. Biomechanical wall stress is a key factor in plaque rupture, as estimated by Laplace’s law^38^. Of utmost importance is to control the blood pressure to prevent rupture from high stress conditions for patients with ACS. Clinically, pressure-controlling medications like beta-blockers are typically recommended for these patients to reduce the coronary circumferential stress^123^. Another example is that elevated WSS and WSS gradient at non-stent treated erosion sites, persisting beyond one year, suggest a continued thrombogenic environment^105^. This suggests that WSS-related factors could be a therapeutic target modulated by the medication or surgery to change the local hemodynamic environment to help prevent future erosion, offering long-term benefits^124^.

## Future Directions and Concluding Remarks

### Mechanical mechanisms of plaque rupture and erosion

Understanding the mechanisms of plaque rupture and erosion are fundamental to develop tailored preventive strategy and treatment option^13,125^. Traditionally, our knowledge on their pathological mechanisms is mainly dependent on ex vivo autopsy studies. Recently, Intravascular OCT imaging technology is emerging as a powerful tool to visualize the distinctive morphological characteristics for reliably detecting these plaques^51^. This imaging modality not only provides a means to monitor the plaques in vivo, but also severs as a basis for constructing image-based computational models to simulate the biomechanical forces^33^. These forces are demonstrated to be important contributors for the development of these pathological processes^30,34^. Therefore, OCT-based computational modeling provides an efficient approach to uncover the mechanical mechanisms of these plaques^13^.

For plaque rupture, there is a general consensus among the experimental and computational studies that rupture usually occurs at the location with high circumferential wall stress^38,98^. Some further claimed that high shear stress also co-localizes with the rupture site^101^. However, mechanical forces are not the only determinant for rupture to happen^17,126^. The inflammation, fibrous cap microstructures and strength also influence the rupture behavior. And there is a still a long way to translate these biomechanical indicators into clinical setting.

Compared to plaque rupture, the biomechanical mechanisms of plaque erosion are far under-investigated. Even if most pathological, clinical and experimental animal studies observed that erosion preferentially occurs to the sites with turbulent flow^127^, they cannot reach an agreement whether low or high shear stress as the dominant mechanical cue to induce endothelium loss and superficial erosion^105^. Great efforts are need to advance our knowledge to the evolution of the plaque erosion. In order to reach a clear firm conclusion, several aspects should be carefully considered and evaluated to resolve the potential discrepancies in the existing literatures: a) There are some inconsistencies exist in these computational studies, like different image modality used for model construction (angiograph or OCT); different patient selection strategy (OCT scan from patients with future erosion, or patients already with erosion). These inconsistencies hindered the head-to-head comparison among these studies. Further efforts should be made to understand the differences among different situations and select the proper data set to investigate mechanisms related to plaque erosion; b) Large-scale computational studies with more patients are needed to identify robust mechanical factors triggering erosion with enough statistical power; c) More animal experiments or other approaches could be carried out to validate the biomechanical findings of plaque erosion studies. The latter two aspects were detailed in the following subsections. Once a clear, firm conclusion on the biomechanical trigger of erosion could be found, it would inspire prosperous molecular and cellular mechanistic studies for a deeper understanding of the mechanisms of erosion.

### Large-scale studies for validation

To obtain a firm conclusion, large-scale studies with enough clinical data are need to reach a strong conclusion with high statistical power. Most of prior biomechanical studies typically had a sample size less than 50, especially for plaque erosion studies. The results of such studies are easily influenced by the patient variability, leading to inconclusive situation^114^. Large-scale studies are recommended to obtain convincing results on the mechanical mechanisms of plaque rupture and erosion in the future analysis.

### Automation of computational simulation procedure for clinical application

Computational modeling approach is widely employed to simulate the biomechanical environment in the coronary plaques for possible mechanistic role of biomechanics in coronary atherosclerosis^33^. However, the biomechanical modeling process is typically time-consuming, especially when performing the FSI analysis^69^. Thus, one prerequisite for performing large-scale computational studies is to automate the computational simulation procedure to reduce labor cost and meet the requirements of early screening purposes. Currently, some advance in CFD analysis has been obtained, the computational solid mechanics model has made less progress partially due to the difficulty in generating the finite element mesh to incorporate the irregular shape of plaque components like lipid core or microcalcification^69^, and solving more complex governing equations. There are multiple components in the atherosclerotic plaques, and it is difficult to obtain their mechanical properties for computational modeling, especially for patient-specific modeling. Furthermore, quantifying the precise boundary conditions for computational solid models, like the supporting forces from the perivascular connective tissue is also challenging. More attempts should be made to overcome these issues if we would like to explore more impacts of solid mechanics in plaque behaviors.

### In vivo animal models or in vitro tissue engineering model of coronary atherosclerosis

It is well-recognized that the atherosclerotic plaques in the animal models are different from the human ones^128^. Even though much progress has been made to create animal models for plaque rupture and erosion, they have limited ability in fully mimicking the natural history of rupture and erosive behavior of plaques^86,127,129^. These challenges pose much difficulty to study the mechanisms to the pathology of these plaque lesions. Another choice is 2D cellular culture model. Given they cannot recapitulate the complex vascular structure, with multiple vascular cells, extracellular matrix, and other substrates in the coronary artery, 2D cellular culture models are often employed to investigate the cellular and molecular mechanisms in atherosclerosis^130^. Recently, Tissue engineering approach has emerged as a valuable in vitro model for studying atherosclerosis^130^. Tissue engineering blood vessel with early atherosclerosis was created, which showed promise to replace the animal models for studying atherosclerotic process^131^. A prototype work has already performed to understand the biomechanical impact of microcalcification on rupture^132^. This approach has advantages over 2D cellular culture model by incorporating the complex interactions between vascular cells and substrate matrix^130^. And it would be easier to control the mechanical environments in the tissue-engineered blood vessel by controlling flow rate or modulating engineered vessel matrix stiffness^133^. Therefore, tissue-engineered approach may hold the potential to validate key mechanical factors that predispose to erosion. Additionally, this approach enables detailed observation of plaque behaviors at the micro-level^134^, like endothelial denudation, which are challenging to detect in clinical trials and animal studies. Nevertheless, this novel bioengineering approach is still in its infancy and requires more investigations.

To conclude, biomechanical stresses play a crucial role in influencing endothelial function, vascular remodeling, plaque development and deleterious behaviors causing ACS. Due to the challenges of directly measuring these stresses in human coronary arteries, computational biomechanical models have become the mainstream approaches to obtain these mechanical data, and should be incorporated with morphological characteristics to investigate the mechanisms to plaque rupture and erosion. High-resolution OCT imagebased finite element models have revealed a significant difference in the biomechanical environments predisposing to fibrous cap rupture or superficial erosion. A deeper understanding of the effects of mechanical forces in these plaque behaviors will further the developments of novel drugs, enhancing diagnostic tools and informing clinical decision-making for tailored management of plaque rupture and erosion.

## Data Availability

No datasets were generated or analysed during the current study.
