# Author Correction: Role of biomechanical factors in plaque rupture and erosion: insight from intravascular imaging based computational modeling

**DOI:** 10.1038/s44325-025-00069-3

**Published:** 2025-06-17

**Authors:** Liang Wang, Yanwen Zhu, Chen Zhao, Akiko Maehara, Rui Lv, Xiaoya Guo, Mingming Yang, Gary S. Mintz, Dalin Tang, Haibo Jia, Bo Yu

**Affiliations:** 1 School of Biological Science and Medical Engineering, Southeast University, Nanjing, 210096 China; 2 State Key Laboratory of Frigid Zone Cardiovascular Diseases (SKLFZCD), Key Laboratory of Myocardial Ischemia, Chinese Ministry of Education, Department of Cardiology of the Second Affiliated Hospital, Harbin Medical University, Harbin, 150086 China; 3 Cardiovascular Research Foundation, Columbia University, New York, NY 10022 USA; 4 Department of Cardiovascular Surgery, Shandong Second Provincial General Hospital, Jinan, 250022 China; 5 School of Science, Nanjing University of Posts and Telecommunications, Nanjing, 210023 China; 6 Department of Cardiology, Zhongda Hospital, School of Medicine, Southeast University, Nanjing, 210009 China; 7 Mathematical Sciences Department, Worcester Polytechnic Institute, Worcester, MA 01609 USA

**Keywords:** Acute coronary syndromes, Biophysical methods

Correction to: *npj Cardiovascular Health* 10.1038/s44325-025-00048-8, published online 16 April 2025

In this article the Competing Interests statement was incorrect and should have been ‘Author Haibo Jia is a Guest Editor at npj Cardiovascular Health. Haibo Jia was not involved in the journal’s review of, or decisions related to, this manuscript.’ The original article has been corrected.

